# Microbial lipid and amino sugar responses to long-term simulated global environmental changes in a California annual grassland

**DOI:** 10.3389/fmicb.2015.00385

**Published:** 2015-05-05

**Authors:** Chao Liang, Jessica L. M. Gutknecht, Teri C. Balser

**Affiliations:** ^1^State Key Laboratory of Forest and Soil Ecology, Institute of Applied Ecology, Chinese Academy of SciencesShenyang, China; ^2^Department of Soil Science, University of Wisconsin-MadisonMadison, WI, USA; ^3^Department of Soil, Water and Climate, University of Minnesota, Twin CitiesMN, USA; ^4^Department of Soil and Water Science, University of Florida, GainesvilleFL, USA

**Keywords:** lipid, amino sugar, microbial biomass, microbial residue, warming, nitrogen deposition, elevated CO_2_, soil carbon stabilization

## Abstract

Global environmental change is predicted to have major consequences for carbon cycling and the functioning of soil ecosystems. However, we have limited knowledge about its impacts on the microorganisms, which act as a “valve” between carbon sequestered in soils versus released into the atmosphere. In this study we examined microbial response to continuous 9-years manipulation of three global change factors (elevated CO_2_, warming, and nitrogen deposition), singly and in combination using two methods: lipid and amino sugar biomarkers at the Jasper Ridge Global Change Experiment (JRGCE). The two methods yielded important distinctions. There were limited microbial lipid differences, but many significant effects for microbial amino sugars. We found that CO_2_ was not a direct factor influencing soil carbon and major amino sugar pools, but had a positive impact on bacterial-derived muramic acid. Likewise, warming and nitrogen deposition appeared to enrich residues specific to bacteria despite an overall depletion in total amino sugars. The results indicate that elevated CO_2_, warming, and nitrogen deposition all appeared to increase bacterial-derived residues, but this accumulation effect was far offset by a corresponding decline in fungal residues. The sensitivity of microbial residue biomarker amino sugars to warming and nitrogen deposition may have implications for our predictions of global change impacts on soil stored carbon.

## Introduction

Within the context of global change, it is recognized that carbon (C) stabilization in soils is of critical importance, and a better understanding of C biogeochemistry is needed (Lal, 2004; Davidson and Janssens, 2006). Because soil C cycling is ultimately the consequence of microbial growth and activity, the mechanistic basis for understanding C decomposition, transformation, and stabilization in soils lies in a detailed understanding of general microbial physiology and activities, which may act as a “valve” between C sequestered in soils versus released into the atmosphere. It has been well established that the dynamics of the terrestrial C pool are heavily influenced by the catabolic and anabolic activities of microorganisms (Balser, 2005; Schimel and Schaeffer, 2012), and that these activities are essential for biogeochemical cycling, climate change, and ecosystem sustainability (Schimel et al., 2007; Bardgett et al., 2008; Liang and Balser, 2011). However, the direct incorporation of microbial residues (microbial cellular components from both living and senesced biomass), into stable soil C pools (specifically those whose turnover time can be on the order of centuries) has received less attention (Liang et al., 2011; Miltner et al., 2012; Lee and Schmidt, 2014).

Microorganisms can be considered responsible for both the formation and turnover of stable soil C. They decrease the stable C pool by the process of decomposition, but also can contribute to it by production and turnover of their biomass (Liang et al., 2011; Miltner et al., 2012). Historically, direct microbial contribution to soil C sequestration has been regarded as low, and has been considered negligible or even ignored in many instances, as active microbial biomass makes up <5% of soil organic matter (Wardle, 1992; Dalal, 1998), and has been reported as <4% of soil organic C (Anderson and Joergensen, 1997). However, this may be misleading as living biomass alone does not properly indicate long-term C dynamics (Potthoff et al., 2008). Microorganisms can utilize easily degradable substrates for biomass synthesis, and residual parts of their biomass accumulate in soils when they turn over. In the iterative process of cell generation, growth and death, microorganisms continuously add to soil stable C pool. Therefore, microbial “necromass” (senesced cell components) rather than standing biomass may be a better indicator of microbial contribution to soil C pools.

Microbial residues are now thought to play a far greater role in the sequestration of C into soil stable C pools than traditionally believed (Kindler et al., 2006, 2009; Simpson et al., 2007a; Miltner et al., 2009; Liang et al., 2011). Microbial necromass can exist as relatively recalcitrant polymers, some of which are resistant to decomposition, and have been suggested as important components of the relatively stable C pool in soils (Guggenberger et al., 1999; Glaser et al., 2004; Liang et al., 2008). In fact, because of their rapid growth and constant turnover, microbial residues accumulate greatly over time, and therefore the contribution of microbial-derived C in soils is potentially quite large (Liang et al., 2011). Recent analytical work also confirms this: using nuclear magnetic resonance analysis, it has been found that microbial components and metabolic products are shown to have similar structures to stable humic substances that qualitatively indicates a significant incorporation of microbial-derived C (Simpson et al., 2007a,b). Further, microbial-derived sugars are stabilized in finer soil over time, as indicated by high ratios of hexose to pentose (Guggenberger et al., 1994; Kiem and Kogel-Knabner, 2003). In sum, microbial residues can result in a net contribution of microbial-derived C to the soil stable C pool. As it is well established that the stable soil organic C pool is the most important for long-term C sequestration (Swift, 2001), research on formation, storage and transport of microbial residues in soils is critical for understanding microbial involvement and control over the stabilization of organic C, and further global C cycling.

To date investigations about potential ecosystem C storage in response to climate change have not been focused on the degree to which soil microbial-derived C persists and changes, but rather have been focused more generally on the transformation of plant-derived C. However, given the potential significance of microbial contribution to, and control over, stable soil C, accurate prediction of the impact of climate change drivers on soil organic C will likely require understanding the response of microbial-derived recalcitrant compounds to a range of environmental factors that affect microbial growth and activities (such as soil water, nitrogen deposition, temperature). While there are studies addressing these independently (Millar et al., 2004; van Groenigen et al., 2007; Zhang et al., 2014), few have explicitly investigated their simultaneous impact.

A detailed understanding of C transformation and sequestration driven by microbial communities can not be obtained by analysis of bulk microbial biomass alone, as incorporation of microbial biomass C into soil organic C does not significantly increase the total C to the soil (Potthoff et al., 2008). Also, measurement of total microbial residues is difficult since reliable differentiation between the C bound in microbial residues and soil extant organic C is still unavailable. Alternatively, biomarker molecules can be used to trace the microbial origin of soil organic C (Boschker and Middelburg, 2002; Joergensen and Emmerling, 2006). Microbial residues contain characteristic amino sugars that can be used as time-integrated biomarkers because of their absence in plants (Amelung, 2001), and their stability against degradation (Nannipieri et al., 1979; Chantigny et al., 1997). Microbial amino sugars have been shown to be a relatively stable fraction of the microbial biomass, and persist after cell death, thus the proportion of total amino sugars to total soil C has been used to characterize the relative contribution of the microbial community to soil C turnover and storage (Guggenberger et al., 1999; Amelung, 2001; Glaser et al., 2004; Joergensen and Emmerling, 2006; Niggemann and Schubert, 2006).

In this study, we quantify living microbial biomass using lipid analysis and microbial residues by amino sugar analysis in a California annual grassland ecosystem continuously exposed for 9 years to elevated CO_2_, water addition, warming, and nitrogen (N) deposition, alone or in combination, at the Jasper Ridge Global Change Experiment (JRGCE) facility. These four factors have widely been shown to impact above ground C dynamics (plant production and turnover; i.e., Dukes et al., 2005), and our intent with the work reported here was to increase our corresponding understanding of their impact on below ground C, using microbial residues as a proxy. The two methods – lipid and amino sugar analysis reflect microbial components with very different turnover times. Lipids (with their rapid turnover following cell death) represent the extant, active community, and amino sugars (which have been shown to persist indefinitely in soil) are reflective of both extant and past soil communities. We propose that by using both methods it is possible to identify nuances of the long-term effects on soil C that would be missed by conventional bulk C analysis (Schmidt et al., 2015). Based on prior work indicating no significant impact of water addition on general microbial community structure (Gutknecht et al., 2012), we chose to focus on CO_2_, temperature, and nitrogen. We hypothesized that above-ground elevated CO_2_ treatment,would have little direct impact on microbial community structure or residues and that temperature and nitrogen treatments would have an effect on microbial residues after 9 years [though the exact direction of change was unclear; both elevated temperature and nitrogen have been shown to increase or decrease microbial stable C (Wixon and Balser, 2009; Wang et al., 2014a,b; Griepentrog et al., 2015)]. More specifically our objectives were to: (1) examine and quantify soil microbial response to 9 years of continuous global environmental changes using methods reflecting two distinct timescales of microbial biomass production and turnover; and (2) explore the mechanism and potential feedbacks of microbial-derived C contribution to soil C storage under global environmental changes.

## Results

### Microbial Lipids

**FIGURE 1:**
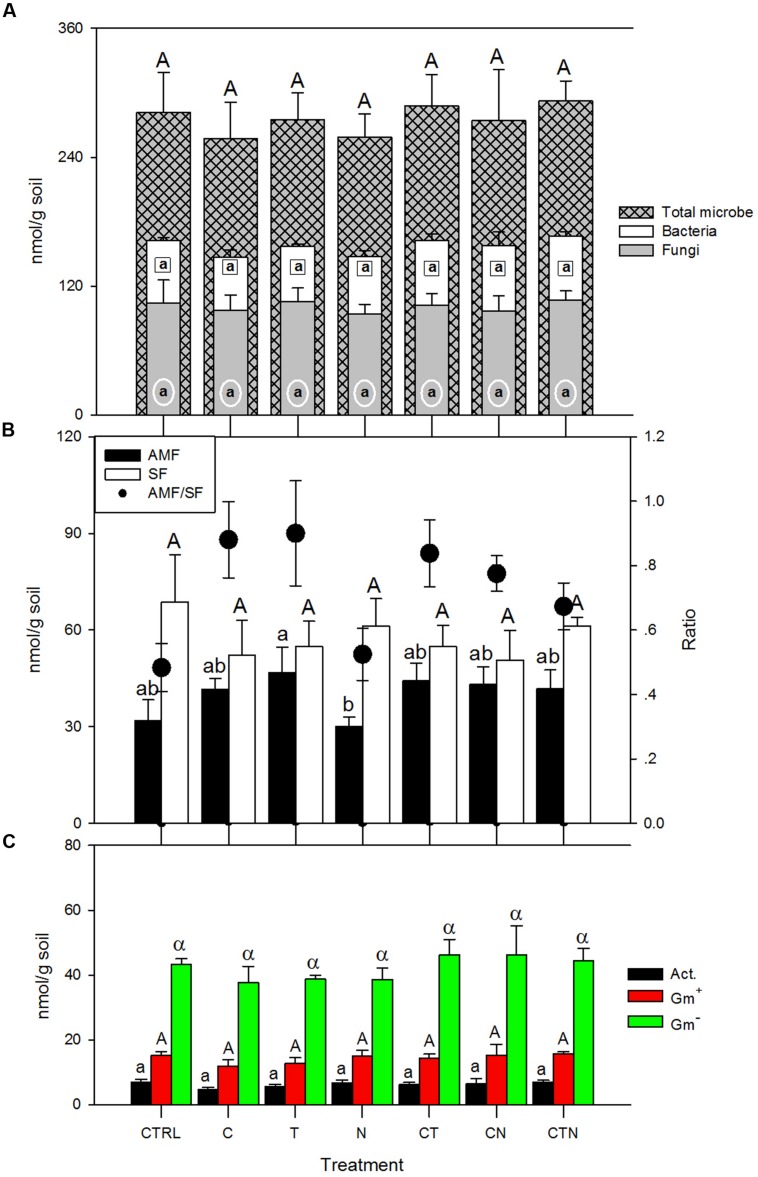
**Lipid guild abundance (nmol/g soil) under single and combined global change treatments. Bars indicate SE of the mean (*n* = 4 or 5).** Bars sharing the same letter are not significantly different (*P* = 0.05, LSD test). CTRL, control; C, elevated CO_2_; T, elevated temperature; N, nitrogen deposition; AMF, arbuscular mycorrhizal fungi; SF, saprotrophic fungi; Gm^+^, gram-positive bacteria; Gm^-^, gram-negative bacteria; Act., actinomycete.

We did not find any statistically significant differences (*P* > 0.05) in fungal, bacterial, or total microbial lipid biomass (nmol/g-soil) among all treatments (**Figure 1A**). General linear model (GLM) analysis showed an insignificant impact of elevated CO_2_, warming and N deposition on general lipid indices (**Table 1**). With regard to more specific microbial group abundance among simulated global change treatments, the N addition treatment had lower levels of arbuscular mycorrhizal fungi (AMF) and higher saprotrophic fungi (SF) compared with other global change treatments, resulting in a significantly (*P* < 0.05) lower AMF/SF ratio for N deposition alone (**Figure 1B**). Gram-positive (Gm^+^) bacteria and actinomycetes tended to show similar patterns among global change treatments as those of SF (**Figure 1C**), with an increase under N deposition and decrease with other factors. In treatments with more than one factor, the abundance of the microbial groups (AMF, SF, Gm^+^, and actinomycete) fell between the values of those in single-factor treatments (**Figure 1**).

**Table 1:** Summary of F-ratios from a general linear model, testing for the integrative effects of treatments on amino sugars, lipids, total carbon and nitrogen after 9-years global change manipulation.

	Treatment					
Variable	C	T	N	C.T	C.N	C.T.N
TC%	0.10	0.00	19.60^∗∗∗∗^	3.14^∗^	1.05	2.20
TN%	0.10	0.00	15.78^∗∗∗∗^	2.99^∗^	0.87	0.53
Total lipids (nmol/g)	0.17	0.73	0.08	0.01	0.02	0.04
Fungal lipids (nmol/g)	0.01	0.88	0.03	0.07	0.00	0.04
AMF lipids (nmol/g)	1.44	2.62	0.01	2.68	0.00	0.15
SF lipids (nmol/g)	1.44	2.62	0.01	2.68	0.00	0.15
Bacterial lipids (nmol/g)	0.86	0.18	0.47	0.25	0.17	0.89
SF to AMF ratio	5.06	0.48	0.65	4.09*	0.07	0.51
Gm^+^ to Gm^-^ ratio	2.78	0.18	0.46	1.79	0.23	0.53
Total AS (ug/g)	0.26	37.36^∗∗∗∗^	1.61	0.78	2.27	5.39^∗∗^
GluN (ug/g)	0.63	40.11^∗∗∗∗^	0.73	0.64	2.63	5.21^∗∗^
GaIN (ug/g)	0.13	21.67^∗∗∗∗^	4.66^∗∗^	0.55	1.31	5.30^∗∗^
MurA (ug/g)	16.59^∗∗∗∗^	0.92	14.2^∗∗∗^	0.16	18.11^∗∗∗∗^	0.43
AS/TC (mg/g)	0.22	32.82^∗∗∗∗^	12.05^∗∗∗^	4.05^∗^	4.68^∗∗^	6.57^∗∗^
GluN/TC (mg/g)	0.47	35.58^∗∗∗∗^	9.40^∗∗∗^	3.69^∗^	4.99^∗∗^	6.59^∗∗^
GalN/TC (mg/g)	0.11	21.10^∗∗∗^	14.81^∗∗∗^	3.04*	3.05*	5.77^∗∗^
MurA/TC (mg/g)	8.32^∗∗∗^	0.39	0.74	0.17	6.53^∗∗^	1.46
GluN/GaIN ratio	0.77	14.41^∗∗∗∗^	14.85^∗∗∗∗^	0.01	1.46	0.28
GluN/MurA ratio	5.78^∗∗^	28.2^∗∗∗∗^	3.76^∗^	0.66	13.01^∗∗∗^	3.00^∗^

*F*-ratio statistic is shown with asterisks indicating the *P*-value level. ^∗^*P* < 0.1; ^∗∗^*P* < 0.05; ^∗∗∗^*P* < 0.01; ^∗∗∗∗^*P* < 0.001. C, elevated CO_2_; T, elevated temperature; N, nitrogen deposition; AMF, arbuscular mycorrhizal fungi; SF, saprotrophic fungi; Gm^+^, gram-positive bacteria; Gm^-^, gram-negative bacteria; AS, amino sugar; GluN, glucosamine; GalN, galactosamine; MurA, muramic acid.

### Microbial Amino Sugars

**FIGURE 2:**
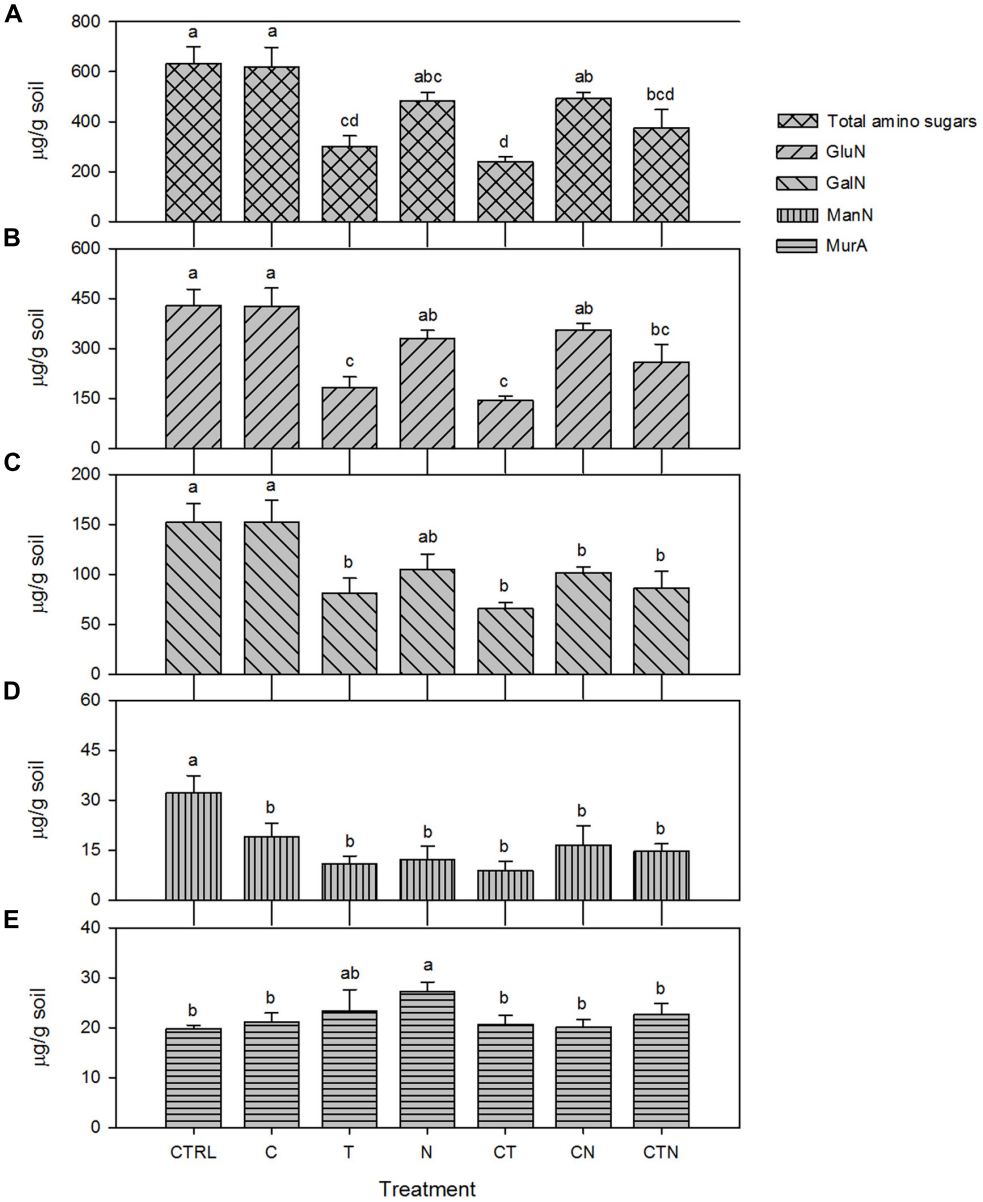
**Absolute abundance in bulk and individual amino sugars (μg/g soil) under single and combined global change treatments.** Bars indicate SE of the mean (*n* = 4 or 5). Bars sharing the same letter are not significantly different (*P* = 0.05, LSD test). CTRL, control; C, elevated CO_2_; T, elevated temperature; N, nitrogen deposition; GluN, glucosamine; GalN, galactosamine; ManN, mannosamine; MurA, muramic acid.

**FIGURE 3:**
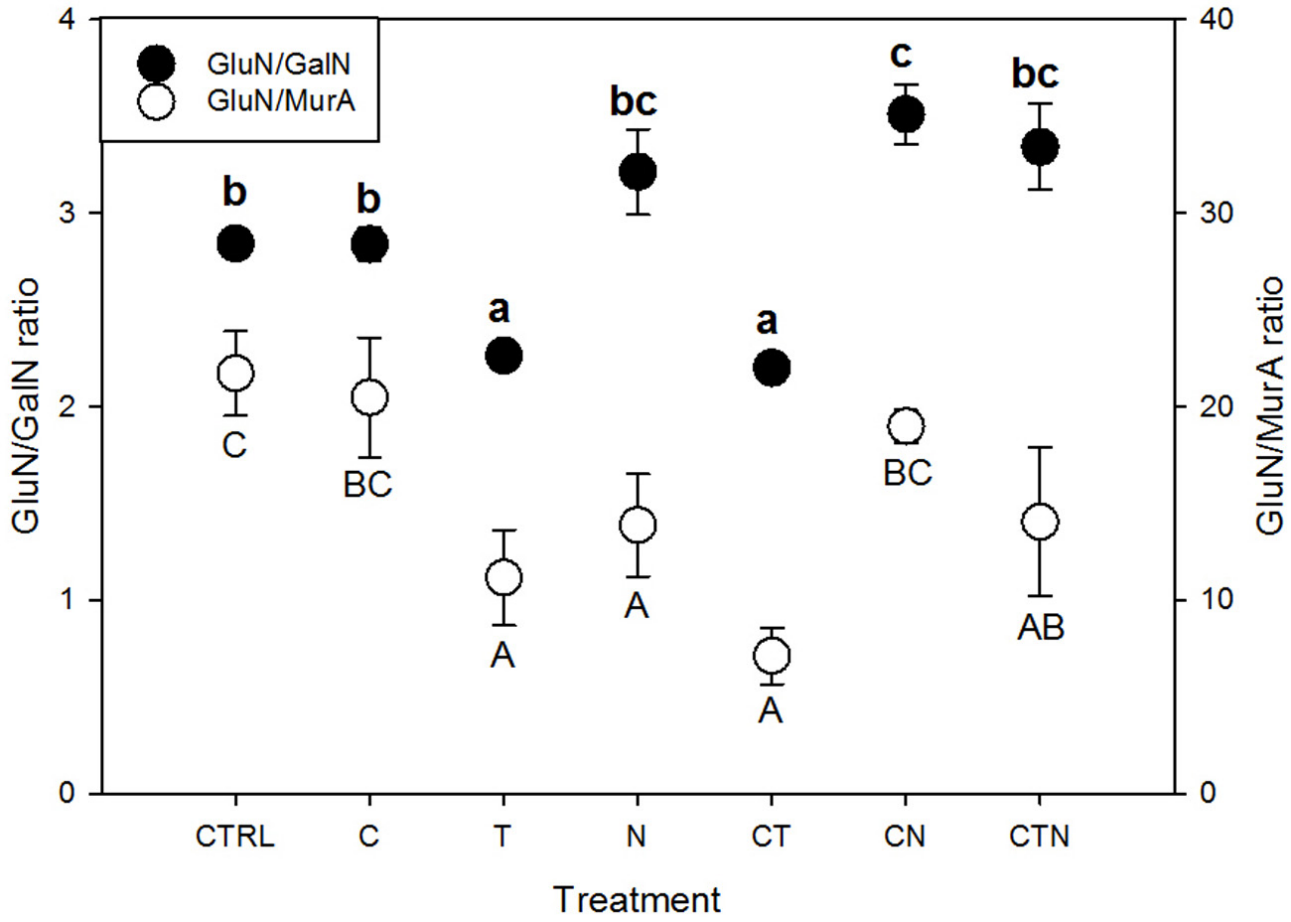
**Amino sugar ratios under single and combined global change treatments.** CTRL, control; C, elevated CO_2_; T, elevated temperature; N, nitrogen deposition; GluN, glucosamine; GalN, galactosamine; MurA, muramic acid. Bars indicate SE of the mean (*n* = 4 or 5). Dots sharing a letter are not significantly different (*P* = 0.05, LSD test).

We found that total amino sugars and the amounts of GluN, GalN, and ManN (μg/g-soil) were depleted, while the bacterial-derived MurA was enriched, under all the JRGCE global change treatments (**Figure 2**). The GLM analysis showed that temperature and N impacted the overall size of the amino sugar pool as well as the proportions of the total amino sugars in the total C pool; CO_2_ had little effect on total amino sugars but did significantly affect the individual amino sugar MurA (**Table 1**). More specifically, temperature and N were both associated with a significant decrease in the total amino sugar abundance by 52.8 and 23.8% respectively (**Figure 2A**), and a significant enrichment in MurA by 18.7 and 37.8% respectively (**Figure 2E**). Elevated CO_2_, on the other hand, showed a suppressive effect in the amount of MurA when in combination with elevated temperature or N addition (**Figure 2E**). The ratios of GluN/GalN and GluN/MurA, a way to measure the amino sugar pattern shift, showed distinct trends. We found that elevated temperature was significantly related to lower GluN/GalN and GluN/MurA ratios, while elevated CO_2_ had no effect. Nitrogen deposition tended to be related to an increased GluN/GalN ratio but a decreased GluN/MurA ratio (**Figure 3**).

### Microbial-Derived C Contribution

**FIGURE 4:**
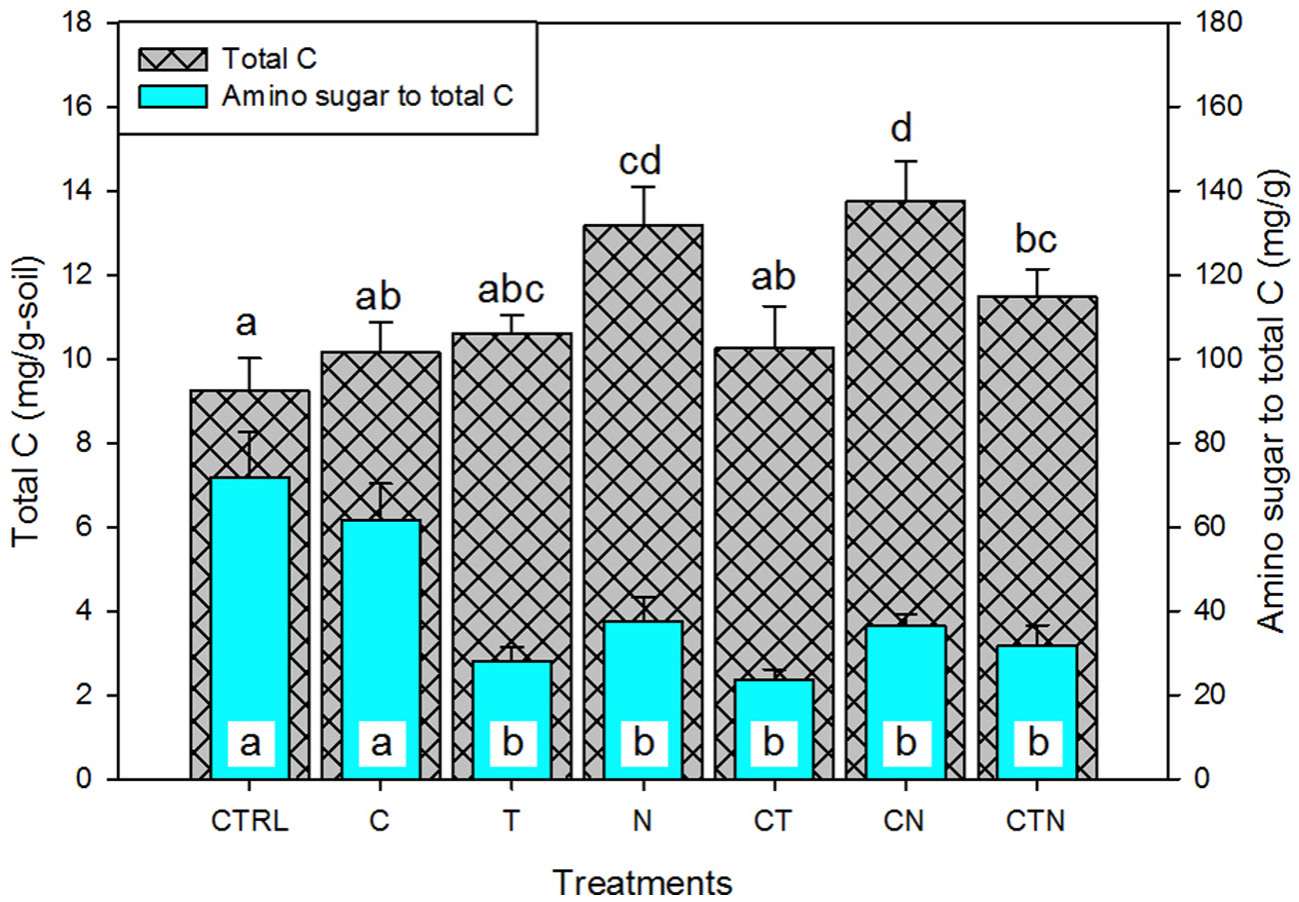
**The effect of single and combined global environmental change treatments on the total carbon pool and the proportion of bulk amino sugars in that pool.** CTRL, control; C, elevated CO_2_; T, elevated temperature; N, nitrogen deposition. Bars indicate SE of the mean (*n* = 4 or 5). Bars sharing a letter are not significantly different (*P* = 0.05, LSD test).

For our second objective, we used the proportion of amino sugars to total soil C as an indication of the relative contribution of microbial-derived residues to stored soil C. Total soil C significantly (*P* < 0.01) increased under N deposition. We found that the amino sugar proportion of total C was not significantly affected by elevated CO_2_, but was negatively affected by warming and N deposition. Also, the negative effects of warming and N deposition were not significantly altered by the addition of CO_2_ as a factor (there was no interactive effect of CO_2_ with these treatments; **Figure 4**). An interactive effect of warming with elevated N and CO_2_ does, however, appear to have a suppressive effect compared with N deposition alone or N deposition coupled with elevated CO_2_ (**Figure 4**).

## Discussion

Our objectives were to investigate response to 9 years of continuous environmental manipulation using two methods of microbial biomarker analysis and to examine contribution of microbial-derived C to soil stable C pools. Changes in microbial lipids reflect an immediate/rapid response to a changed environment, and amino sugars reflect cumulative long-term change. We used lipids and amino sugars simultaneously to study the responses of microorganisms under simulated global environmental changes, and to evaluate the role of microbial ecology in soil C dynamics. Through these simultaneous measurements, we also assess and compare potential long-term sensitivity of soil C to global change.

### Responses to Global Change Treatments

#### Microbial Biomass (Lipids)

We found that total living microbial biomass (lipid abundance) did not differ significantly under any treatment. Changes in microbial biomass under global change scenarios could be important, since microbial biomass has been known as an early indicator of changes in total soil organic C (Powlson et al., 1987). Past studies have shown inconsistent trends for microbial biomass under elevated CO_2_, warming, or N deposition. Reported responses vary in direction and magnitude, and much uncertainty exists on whether microbial biomass will increase, decrease or remain the same under elevated CO_2_ (Zak et al., 2000), warming (Zhang et al., 2005; Frey et al., 2008; Bell et al., 2010) or N deposition (Treseder, 2008). In addition to various mechanisms that contribute to observed microbial abundance, the inconsistency among studies could be a result of high variations in the analysis of microbial living biomass, or in quickly changing dynamics of the living microbial biomass. However, despite the lack of biomass response in this study, microbial community structure did differ in some ways after 9 years of continuous treatment. In particular, fungal community composition appeared altered (AMF to SF ratio). This is aligned with results from Rillig et al. (2002) and Treseder (2004), who found that AMF abundance increased under elevated CO_2_ and soil warming. In contrast, AMF abundance here did not significantly change under N fertilization compared with the control (**Figure 1B**). Two mechanisms have been proposed regarding the N addition influence on AMF: (1) increased N has a negative effect on AMF abundance as AMF become C-limited when N is more available and plants correspondingly reduce allocation of C to mycorrhizal association (Treseder, 2004; van Diepen et al., 2007); (2) AMF proliferation following N addition has also been reported in some ecosystems, possibly because of extreme N-limitation and an increased plant recruitment of AMF (Anderson and Liberta, 1992; Treseder and Allen, 2002). Here, we did not find a significant difference in AMF abundance compared with the control, possibly indicating that multiple mechanisms were involved, or that after 9 years the fungal communities have acclimated to the new level of N-availability.

#### Microbial Residues (Amino Sugars)

We found that total amino sugars, GluN, GalN, and ManN were significantly depleted in warming and N deposition treatments after 9 years, but the MurA was in greater abundance overall. This indicates that 9 years of warming and elevated N significantly reduced the amount of total microbial residues and fungal residues, but augmented bacterial residues. In spite of the differing effects by warming and N deposition on fungal and bacterial residues, they both consistently indicate that N addition has a generally positive effect, and warming has a generally negative effect on microbial residue accumulation. Although the complexity of soil processes makes straightforward interpretations of microbial residue dynamics difficult, we believe that the treatment-induced changes both directly and indirectly influence microbial growth, death and residue decay. The N addition-induced enhancement of plant growth at the JRGCE (Zavaleta et al., 2003; Dukes et al., 2005) could increase C inputs to the soil as litter and root exudation, which may directly benefit microbial growth (Treseder and Allen, 2002). However, plants may also compete with microbes for limited nutrients, which may hamper microbial growth due to nutrient limitation (Treseder, 2004). The treated plots (not N amended plots) at Jasper Ridge are likely a nutrient-limited environment (Menge and Field, 2007; Gutknecht et al., 2010), and plant competition likely limits microbial growth, biomass production, and accumulation. In the case of the warming treatment, changes in microbial physiology may explain the decline in the fraction of assimilated C that is allocated to microbial growth (Allison et al., 2010). In addition, the dominant amino sugars might be decomposed as a type of N nutrient for plant and microbial growth under warming and extreme nutrient-limited conditions. The distinct increase in MurA under treatments could be explained by faster bacterial life cycles associated with faster turnover rate, or a bacterial community shift from Gm^-^ to Gm^+^ bacteria which contain thick murein layers (Eudy et al., 1985; Kogel-Knabner, 2002).

Not surprisingly, there was no significant relationship between elevated CO_2_ and total amino sugars and GluN suggesting that elevated CO_2_ does not influence total microbial residues and fungal residues in soil (**Table 1**, **Figures 2A,B**). In addition, we only found a slight additive influence of elevated CO_2_ coupled with other global change factors (**Figures 2A,B**), which is in accord with the previous work on lipid dynamics over time (Gutknecht et al., 2012). In contrast, elevated CO_2_ was related to moderately higher bacterial-derived MurA compared to the control, suggesting that elevated CO_2_ may contribute to an increased concentration of bacterial-derived residues. In line with this finding, Drigo et al. (2007) found that bacterial community structure in the rhizosphere was most affected by elevated CO_2_, whereas fungal community structure was less influenced.

Direct effects of elevated CO_2_ on soil microbial communities could be expected to be negligible since the CO_2_ concentration in the soil pore space is much higher than the atmospheric level above the soil. However, elevated CO_2_ can indirectly affect soil microbial communities via plant metabolism and root secretion (Drigo et al., 2008), decreased evapotranspiration (Ainsworth and Long, 2005). The Jasper Ridge site, as with typical grassland systems, has a high volume of rhizosphere soil, which was dominated by Gm^-^ bacteria (**Figure 1C**). The increase in MurA under elevated CO_2_ may thus be a result of indirect plant “fertilization,” increased levels of labile C and nutrient excreted into the rhizosphere leading to higher Gm^-^ bacterial growth and turnover.

Variation in amino sugar ratios can be used as a qualitative indicator of time-integrated compositional changes in the soil microbial community (Guggenberger et al., 1999; Glaser et al., 2004). We found that the abundance of GalN was different from MurA among treatments, and thus ratios of GluN/GalN and GluN/MurA showed different tendencies, especially under N deposition where MurA was significantly increased. We suggest using the GluN/GalN ratio to describe overall amino sugar accumulation patterns (long-term microbial turnover), and the GluN/MurA ratio to understand the relative long-term contribution to soil C by fungi versus bacteria.

### Evaluation of Rapid versus Long-Term Responses of Soil Microbes

Another interest of ours was to compare the use of the two microbial methods, each indicating something distinct about soil C. Precise prediction of microbial response to global changes has proven difficult within a specific ecosystem due to the large uncertainty regarding microbial community (Treseder et al., 2012). This could be due both to method limitations and limitations due to the timing and frequency of sampling. A single sampling of microbial biomass may not reveal microbial response to prolonged global changes, as rapid and ephemeral changes in the living microbial community may confound the perceived treatment effects. Another limitation is that the standing biomass of microbes does not necessarily reflect microbial contribution to soil C storage, since microbial-derived residues in nascent humic substances could have increased even while standing living biomass remains constant or decreases (Potthoff et al., 2008). It is reasonable to expect that the different microbial community responses to simulated global changes in our study should sustain microbial residue accumulations to a different degree, and the effects could be further amplified over time; while that measures of standing biomass, for instance lipids, may not reveal these long-term changes in microbial contributions to soil C (**Table 2**). As microbial cell walls appear more stable and turn over more slowly than living microbial biomass (Guggenberger et al., 1999; Amelung, 2001; Glaser et al., 2004), microbial cell wall amino sugars provide a more useful indicator of chronic, long-term soil microbial responses and microbial necromass contributions to changes in soil C storage.

**Table 2:** Summarized dynamics of lipids and amino sugars under 9-years simulated global changes in JRGCE.

	Lipid			Amino sugar				
	Total lipids	Fungal lipids	Bacterial lipids	Total amino sugars	Glucosamine	Muramic acid	Proportions of amino sugars in soil C
Elevated CO_2_	No change	No change	No change	No change	No change	Increase	No change
Warming	No change	No change	No change	Decrease	Decrease	Increase	Decrease
N deposition	No change	No change	No change	Decrease	Decrease	Increase	Decrease

### Microbial Residue Amino Sugar Contribution to Soil C Storage

Our second goal with this research was ultimately to go beyond describing microbial responses following 9 years of manipulation and assess the potential significance of a given response for below ground C storage. Toward that end, the amino sugar proportion of total soil C may be used to indicate microbial residue accumulation, or microbial-derived C contribution to soil C sequestration over time (Liang and Balser, 2012). In addition to examining absolute amino sugar amounts (indicating extant and past microbial biomass and coarse community structure), it is interesting to consider how the proportion of amino sugars to soil total C was altered by the global change manipulations. In the Jasper Ridge grassland ecosystem, elevated CO_2_ had no significant effect on total soil C or its proportional amino sugar content. However, under increased nitrogen and warming, total soil C showed two responses: 9-years of N deposition significantly increased soil total C storage, while warming did not statistically alter the total soil C pool (**Figure 4**). In contrast, the amino sugar proportion of total soil C was lowered under both warming and N deposition (**Table 1**; **Figure 4**). Thus microbial residues appeared to decline in contribution to total soil C under warming and N deposition compared with other C-containing compounds. This has potential implication for long-term C stabilization or sequestration under warming or N deposition. The decline in contribution of microbial C may reflect differing underlying impacts of the two environmental changes on microbial dynamics (Liang and Balser, 2012). Temperature is a global modulator affecting respiration, enzyme activity, and membrane fluidity – all living organisms must respond in some way to a change in temperature. Increased temperature has been shown to correlate to a decline in microbial biomass (Gutknecht et al., 2012), and this decline may be responsible for the lowered contribution of amino sugars-C to total soil C. Nitrogen is a resource, not a modulator. Organisms compete for it, and it is often related to increased overall biomass. Thus the lower microbial amino sugars-C relative to total soil C in this case is likely due to an increase in newer plant C when N constraints on plant production are alleviated rather than a decline in microbial contribution.

This highlights the need to look at soil C as more than a bulk measure, as is also being recognized in other research efforts (Poirier et al., 2005; Schmidt et al., 2015). Assuming soil C is a single homogeneous pool ignores the fact that separate fractions have different origins or turnover time (Trumbore, 1997, 2000). The soil stable C pool is critical in the role of soil as a C sink within the global C cycle, as even a small change in the stable C pool could have large consequences for climate change (Rustad et al., 2000). In this study, microbial residue biomarker analysis allowed us to separate organic C of microbial origin from other fractions. While their percentage of total C is relatively small, microbial amino sugars may act as a canary in the mineshaft for soil C – indicating possible vulnerabilities or changes in long term stability. The sensitivity of microbial residues to warming and N deposition observed in this study may prove important in our predictions of global change impacts on soil stored C.

## Conclusion

The overall purpose of this study was to explore soil microbial communities and potential responses of stable soil C to long-term simulated global environmental changes. We used microbial biomarkers, lipids and amino sugars, to investigate microbial responses and contribution of microbial residues. Amino sugars, as compared to lipids, provide a more useful indicator of long-term soil microbial responses, and reflect microbial residue contributions to soil C storage under chronic simulated global environmental changes. We found that an environmental modulator (temperature) and a nutrient resource (nitrogen) differed in their impact on microbial residue accumulation. Warming and N deposition both resulted in significantly depleted total microbial residues, and enriched bacterial residues (though offset by the corresponding decline in fungal residues).

This study highlights the power of the amino sugar analysis by directly comparing potential microbial responses on short and long time scales (using lipid and amino sugar biomarkers as reflective of these timescales, respectively) in a grassland after continuous 9-years manipulation of global multifaceted factors. We suggest that single time point microbial lipid measurements may not reveal the long-term microbial contribution to soil C in the same way that analysis of microbial amino sugars can. The study also highlights the sensitivity of microbial residues in the soil C stock in the context of warming and N deposition. We suggest the dynamics of microbial residues should be incorporated into current soil C research and that integration may substantially improve our predictions of global change impacts on soil stored C.

## Materials and Methods

### Site Description

The JRGCE contains manipulations of each of the following global change factors: (1) atmospheric CO_2_ concentration (ambient and 700 ppm), (2) temperature (ambient and +1 degree Celsius at the soil surface), (3) N deposition (ambient and 7 g N m^-2^y^-1^), 4) water addition (ambient and augmented to twice ambient), with all factors crossed in a complete full factorial design (Shaw et al., 2002; Zavaleta et al., 2003). In this study we did not include samples from the water addition plots. The JRGCE was started in 1998 to test future global change scenarios for this region (Hayhoe et al., 2004). The site is located in the eastern foothills of the Santa Cruz Mountains at the Jasper Ridge Biological Preserve in the San Francisco Bay area. The region experiences a Mediterranean climate, and is generally dominated by annual non-native grasses *Avena barbata* and *Avena fatua* (Zavaleta et al., 2003). The soil at the JRGCE is a loam texture with a pH ranging from 6.5 to 7.0, ∼3% organic matter and a cation exchange capacity of 9.5 meq 100 g soil^-1^. Soils were sampled on 16–17 April, 2007 from the JRGCE, corresponding with annual peak plant biomass, 9 years following the start of the experiment. Four soil cores (4 cm diameter by 15 cm depth) were taken from each experimental plot, 10-g sub-samples from the homogenized cores were frozen immediately, shipped to the University of Wisconsin – Madison, and freeze dried for microbial analysis. Other sub-samples were air dried for total C and N analysis. Total soil C and N data were determined by combustion analysis and provided by the JRGCE facility support staff.

### Microbial Analysis

We assayed the soils for microbial amino sugars and lipids. We determined four amino sugars [glucosamine, galactosamine (GalN), mannosamine (ManN), and muramic acid] by gas chromatograph (GC) after their conversion to aldonitrile acetates according to the protocol of Guerrant and Moss (1984) and Zhang and Amelung (1996), as modified by Liang et al. (2012). Briefly, ∼1 g soil samples were hydrolyzed with 6M HCl at 105°C for 8 h, and then the solution was filtered and purified by neutralization. After drying the supernatant, methanol was used to wash amino sugars from the residues, transferred to 3 mL vials, and then evaporated to dryness on a Labconco 79000 RapidVap (Labconco Co., Kansas City, MO, USA). The residues were re-dissolved in 1 mL distilled deionized water, lyophilized overnight, and then processed for aldononitrile acetate derivatization. To prepare the aldononitrile derivatives, the amino sugars were dissolved in a derivatization reagent consisting of hydroxylamine hydrochloride (32 mg mL^-1^) and 4-(dimethylamino) pyridine (40 mg mL^-1^) in 4:1 pyridine-methanol (v/v), and heated to 75–80°C for 35 min. Then, 1 mL acetic anhydride was added, and the solution was reheated for 25 min for acetylation. After cooling, 1.5 mL dichloromethane and 1 mL 1M HCl were successively added and the mixture was vortexed to transfer the amino sugar to organic phase. The solution was washed thrice with 1 mL deionized H_2_O to remove excess anhydride. In the last washing step, the aqueous phase was removed as completely as possible. Finally, the organic phase was dried at 45°C in a Labconco 79000 RapidVap, and re-suspended in 300 μL ethyl acetate-hexane (1:1) for GC analysis. Separation of amino sugar derivatives was carried out on an Agilent 6890 GC (Agilent Technologies, Wilmington, DE, USA) equipped with a J&W Scientific Ultra-2 column (25 m by 0.2 mm by 0.33 μm) and flame ionization detector (FID). Sample extracts (2 μL) were injected onto the column using H_2_ as the carrier gas at a constant flow rate of 0.4 mL min^-1^. The GC inlet was set to 250°C and operated in split mode with a 30:1 ratio. The individual amino sugar derivatives were identified by comparing their retention time with those of authentic standards. Quantification was gained relative to the internal standard myo-inositol, which was added to the samples prior to purification, and the recovery standard *N*-methylglucamine was added before derivatization. The recovery standard was used to assess the reliability of derivatization step. Glucosamine (GluN, made by fungi and bacteria) and muramic acid (MurA, made by bacteria) are both particularly useful biomarkers. Fungal GluN is often the most abundant amino sugar found in soils, while MurA is uniquely derived from bacterial peptidoglycan (Amelung, 2001; Joergensen and Emmerling, 2006). The others, GalN and mannosamine, are less useful due to questions about their origins (Coelho et al., 1997; Amelung, 2001; Engelking et al., 2007).

We used a hybrid procedure of phospholipid fatty acid (PLFA) and fatty acid methyl ester (FAME) analysis to assay microbial community structure (Kao-Kniffin and Balser, 2007). We extracted lipids from ∼3 g freeze-dried sub-samples using a phosphate buffer, chloroform and methanol (0.9:1:2) extraction solution. Samples were extracted twice, and then the supernatant were combined together. After phase separation overnight, the organic phase isolated and evaporated to dryness using a RapidVap (LabConco, Kansas City, MO, USA). We carried out FAME analysis as described by Microbial ID Inc on the dried organic residue. Lipids were saponified, and then subjected to alkaline methanolysis. Lipids were isolated from the samples in a hexane extraction. We analyzed extracts using a Hewlett-Packard Agilent 6890 GC (Agilent Technologies, Wilmington, DE, USA) equipped with an Agilent Ultra-2 (5% phenyl)-methylpolysiloxane capillary column (25 m by 0.2 mm by 0.33 μm) and FID. Lipid peaks were identified by MIDI peak identification software (“Sherlock microbial identification system,” MIDI Inc., Newark, DE, USA). Two internal standards, 9:0 nonanoic methyl ester and 19:0 nonadecanoic methyl ester, were used as internal standards to convert fatty acid peak areas to the absolute abundance at nmol/g-soil. We quantified the abundance of different microbial groups in each treatment using the abundance of lipids in chemically similar ‘guilds’. Microbial biomass was represented by the sum of all identifiable fatty acids (detectable at >0.05% and C number <20). Fungal, bacterial and actinomycetal biomass were represented by summing all representative PLFAs for each group. In specific, we used the sum of 14:0iso, 15:0anteiso, 15:0iso, 16:0iso, 17:0anteiso, and 17:0iso to indicate Gm^+^ bacteria, and the sum of 16:1ω7c, 16:1ω9c, 17:0cyclo, 17:1ω8c, 19:0cyclo and 18:1ω7c to indicate Gm^-^ bacteria. The sum of 10Me18:0; 10Me17:0; 10Me16:0 represents actinomycete. AMF is indicated by 16:1ω5c and SF by the sum of 18:1ω9c and 18:2ω6c.

### Statistics

In order to test the global change effects, we took single and combined treatments as the different levels of global change, and inferred the effects with one-way ANOVA for the abundance of individual and total lipids and amino sugars, ratios of lipids and amino sugars, and soil total C. A *post hoc* separation of means by LSD was performed in the cases where main effects were significant at *p* < 0.05. We used a GLM to test the integrative effects of elevated CO_2_, warming, N deposition and their interactions on microbial lipids and amino sugars. Statistical analyses were performed with the SPSS (SYSTAT Software, Inc.) software for Windows, and figure preparations were accomplished using Sigma Plot (SYSTAT Software, Inc.).

## Conflict of Interest Statement

The authors declare that the research was conducted in the absence of any commercial or financial relationships that could be construed as a potential conflict of interest.
